# Author Correction: Tunable metasurfaces via the humidity responsive swelling of single-step imprinted polyvinyl alcohol nanostructures

**DOI:** 10.1038/s41467-022-34566-1

**Published:** 2022-11-09

**Authors:** Byoungsu Ko, Trevon Badloe, Younghwan Yang, Jeonghoon Park, Jaekyung Kim, Heonyeong Jeong, Chunghwan Jung, Junsuk Rho

**Affiliations:** 1 Department of Mechanical Engineering, Pohang University of Science and Technology (POSTECH), Pohang, 37673 Republic of Korea; 2 Department of Chemical Engineering, Pohang University of Science and Technology (POSTECH), Pohang, 37673 Republic of Korea; 3 POSCO-POSTECH-RIST Convergence Research Center for Flat Optics and Metaphotonics, Pohang, 37673 Republic of Korea; 4 National Institute of Nanomaterials Technology (NINT), Pohang, 37673 Republic of Korea

**Keywords:** Metamaterials, Nanophotonics and plasmonics, Sensors, Polymers, Surface patterning

Correction to: *Nature Communications* 10.1038/s41467-022-32987-6, published online 21 October 2022

The original version of this Article contained an error in Fig. 5, in which the schematic arrows were incorrectly numbered. This has been corrected in both the PDF and HTML versions of the Article.

