# Supplementary material for: Tunable metasurfaces via the humidity responsive swelling of single-step imprinted polyvinyl alcohol nanostructures
Source: Nat Commun. 2022 Oct 21;13:6256. doi: 10.1038/s41467-022-32987-6 (PMC9587293; doi:10.1038/s41467-022-32987-6)
Supplement: Supplementary file 1 — Supplementary Information [file 41467_2022_32987_MOESM1_ESM.pdf]

Supplementary Information

Tunable metasurfaces via the humidity responsive swelling of single-step imprinted polyvinyl alcohol nanostructures

Byoungsu Ko[1,†], Trevon Badloe[1,†] Younghwan Yang[1,†], Jeonghoon Park[1], Jaekyung Kim[1], Heonyeong Jeong[1], Chunghwan Jung[2], Junsuk Rho[1,2,3,4*]

[1]Department of Mechanical Engineering, Pohang University of Science and Technology (POSTECH), Pohang 37673, Republic of Korea

[2]Department of Chemical Engineering, Pohang University of Science and Technology (POSTECH), Pohang 37673, Republic of Korea

[3]POSCO-POSTECH-RIST Convergence Research Center for Flat Optics and Metaphotonics, Pohang 37673, Republic of Korea

[4]National Institute of Nanomaterials Technology (NINT), Pohang 37673, Republic of Korea

[†]These authors contributed equally to this work

[*]E-mail: jsrho@postech.ac.kr

This PDF file includes

Supplementary Note 1. Thickness of spin-coated 3wt% and 5wt% PVA thin films
Supplementary Note 2. The swelling characteristics of 3wt% PVA thin films
Supplementary Note 3. Transparency of PVA thin-films
Supplementary Note 4. Ellipsometry model for measurement of PVA refractive index
Supplementary Note 5. Refractive index calculation using effective medium theory
Supplementary Note 6. Uniformity of PVA films
Supplementary Note 7. PVA viscosity for NIL
Supplementary Note 8. Resolution of nanostructured PVA with NIL
Supplementary Note 9. Experimental confirmation of the non-toxic nature of the PVA metasurfaces
Supplementary Note 10. Reflectance spectra depending on geometry and sizes of PVA nanostructures
Supplementary Note 11. Theory of PB-phase
Supplementary Note 12. PVA swelling simulations
Supplementary Note 13. Apparent unevenness in the reflected color
Supplementary Note 14. Measured relative humidity of human breath
Supplementary Note 15. Calculated and measured reflectance spectra
Supplementary Note 16. Measured absorption characteristics
Supplementary Note 17. Increased noise in the holographic image due to high RH
Supplementary Note 18. Measurement of the decryption threshold for the Pt coated PVA metasurfaces
Supplementary Note 19. Robustness of the PVA metasurfaces

Supplementary Note 1: Thickness of spin-coated 3wt% and 5wt% PVA thin films

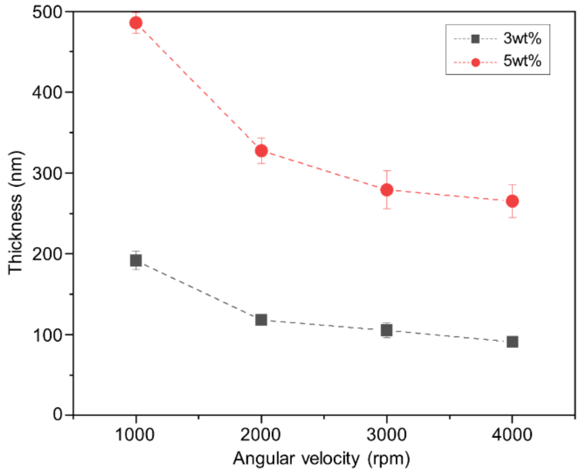

Supplementary Figure 1. Measured spin-coated polyvinyl alcohol (PVA) film thickness. The measured thickness of PVA films spin-coated from 1,000 rpm to 4,000 rpm for PVA concentrations of 3wt% (black) and 5wt% (red). Error bars: one standard deviation.

At the same angular velocity, the thickness of the spin-coated PVA film depends on the concentration. Higher concentration of PVA leads to thicker films, with an increase in the volume of PVA molecules.

Supplementary Note 2: The swelling characteristics of 3wt% PVA thin films

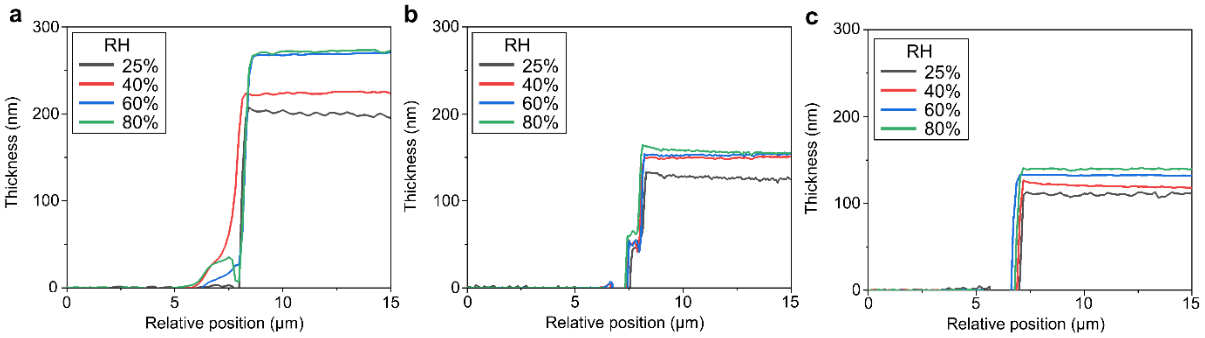

Supplementary Figure 2. 3wt% polyvinyl alcohol (PVA) film thickness at different relative humidity (RH). The thickness of 3wt% PVA films spin-coated at **(a)** 1,000 rpm, **(b)** 2,000 rpm, and **(c)** 3,000 rpm measured using atomic force microscope.

Supplementary Table 1. Measured thickness of spin-coated polyvinyl alcohol (PVA) thin films.

RH (%)	Angular velocity (rpm)		
	3,000	2,000	1,000
	Thickness (nm)		
**25**	109	121	200
**40**	119	149	224
**60**	132	152	268
**80**	139	156	271
**Thickness increase (%)**	**27.5**	**28.9**	**35.5**

Depending on the initial thickness of the spin-coated PVA film, the total increase of the thickness during swelling is modulated due to the volume of PVA in the film. Lower angular velocities produce thicker films that show larger amounts of swelling, up to 35.5% for a film deposited at 1,000 rpm compared to 27.5% increase for the film deposited at 3,000 rpm.

Supplementary Note 3: Transparency of PVA thin-films

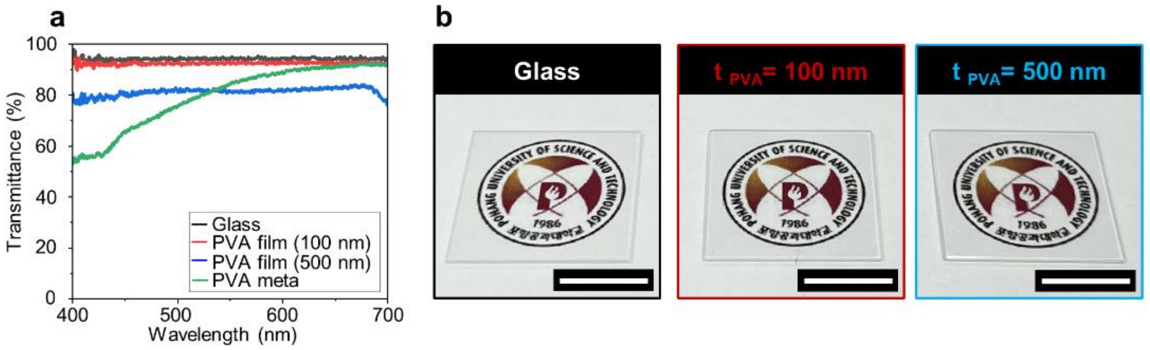

Supplementary Figure 3. Transmittance spectra of polyvinyl alcohol (PVA) film & metasurfaces. (a) The measured transmittance comparison of bare glass, thickness variation PVA films, and presence of metasurface array. **(b)** Photograph of non-coated bare glass (black), the PVA films spin-coated at 3,000 rpm with 3wt% (red), at 1,000 rpm with 5 wt% (blue).

The measured transmittance spectra show the PVA polymer is nearly transparent in visible frequency. Significantly, the difference in transmittance in the PVA film thickness variation. It originates from a degree of surface roughness, which promotes higher scattering through the increased granules[1]. In other words, it elevates the reflectance, thereby diminishing the transmittance. The surface uniformity photograph of PVA films depends on the relation between the spin-curve and concentration will be discussed in Supplementary Note 6.

Supplementary Note 4: Ellipsometry model for measurement of PVA refractive index

Refractive index of polyvinyl alcohol (PVA) films were measured with ellipsometry, and the measured data is fitted using the Cauchy dispersion model, which is described as:

$$n = \mathrm{A} + \frac{\mathrm{B}}{\lambda^2} + \frac{\mathrm{C}}{\lambda^4} \tag{1}$$

where, n is refractive index of PVA; λ is the wavelength; and A, B, C are coefficients that can be determined by fitting the equation to measure data. The measured n is described with the fitted coefficients of A = 1.502, B = 0.00437, and C = 0.0000893. Root mean square error between measured and fitted data is 8.927.

Supplementary Note 5: Refractive index calculation using effective medium theory

The Maxwell Garnett mixing formula is used to estimate the refractive index of polyvinyl alcohol (PVA) when 35.5% of the volume is occupied by water (n = 1.33)[2].

$$\epsilon_{\mathrm{s\_PVA}} = \epsilon_{\mathrm{PVA}} \frac{\epsilon_{\mathrm{PVA}} + \frac{1+2f}{3}(\epsilon_{\mathrm{water}} - \epsilon_{\mathrm{PVA}})}{\epsilon_{\mathrm{PVA}} + \frac{1-f}{3}(\epsilon_{\mathrm{water}} - \epsilon_{\mathrm{PVA}})} \tag{2}$$

where, $\epsilon_{\mathrm{s\_PVA}}$, ϵ_{PVA}, and $\epsilon_{\mathrm{water}}$ are the complex permittivities of the swollen PVA, PVA, and H_2O, respectively, and f is the filling fraction of the water f is set to 0.355 to match the measured maximum swelling ratio of 35.5%.

Supplementary Note 6: Uniformity of PVA films

The uniformity of polyvinyl alcohol (PVA) films depends on the concentration and the angular velocity of the spin coating process. When deposited below 1,500 rpm the films that are produced are not completely dry, even when using low concentration 3wt% PVA aqueous solution[3]. Therefore, the uniformity of the surface of the film is lower, which can be seen in Supplementary Fig. 4 for films deposited at 1,000 rpm. A minimum of 2,000 rpm is recommended for forming films with a higher concentration of PVA solution. Here, to produce films that are uniform enough for nanoimprint lithography (NIL), an angular velocity of 3,000 rpm is used throughout.

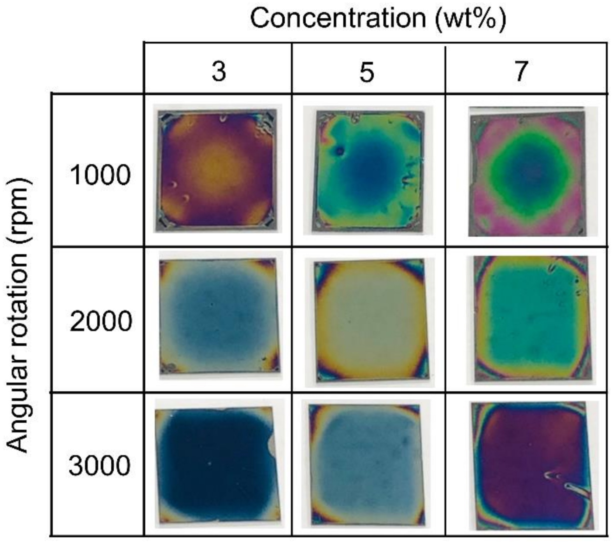

Supplementary Figure 4. Spin-coated polyvinyl alcohol (PVA) films on 2×2 cm^2 silicon substrates.

Supplementary Note 7: PVA viscosity for NIL

We experimentally verify that the 3wt% and 5wt% polyvinyl alcohol (PVA) solution can be used to successfully replicate nanopatterns. Both concentrations of PVA allow for a complete transfer of the pattern to the target substrate. As the concentration of PVA increases over 5wt%, however, the viscosity of the solution increases, limiting its use in one-step nanoimprint lithography (NIL), as can be seen in Supplementary Fig. 5c, the pattern is not successfully transferred to the substrate as the PVA solution sticks to the soft mold due to the increased viscosity. Therefore, we choose to use a concentration of 3wt% throughout.

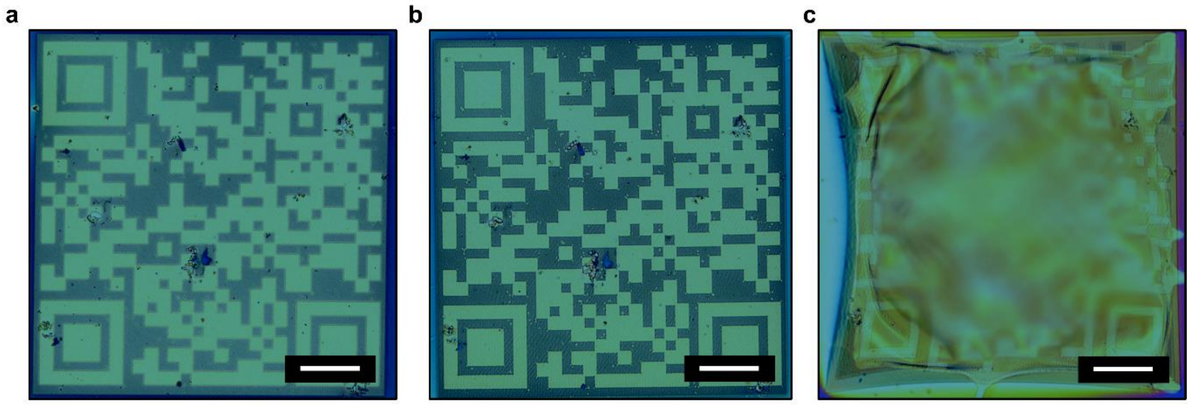

Supplementary Figure 5. Effect of polyvinyl alcohol (PVA) concentration on nanoimprint lithography (NIL) quality. One-step nanoimprinted QR code fabricated using **(a)** 3wt%, **(b)** 5wt%, **(c)** 7wt% PVA. Scale bars: 50 µm.

Supplementary Note 8: Resolution of nanostructured PVA with NIL

We verify the resolution of nanoimprint lithography (NIL) with polyvinyl alcohol (PVA) for the production of nanoscale structures that can be used for optical metasurfaces by printing various grating and pillar structures (Supplementary Fig. 6). We are able to replicate gratings with widths ranging from 50 to 200 nm, and pillars with diameters ranging from 150 to 850 nm, with a height of 500 nm by PVA NIL on silicon substrates. The minimum width of grating is 50 nm, demonstrating a high-aspect ratio of 10.

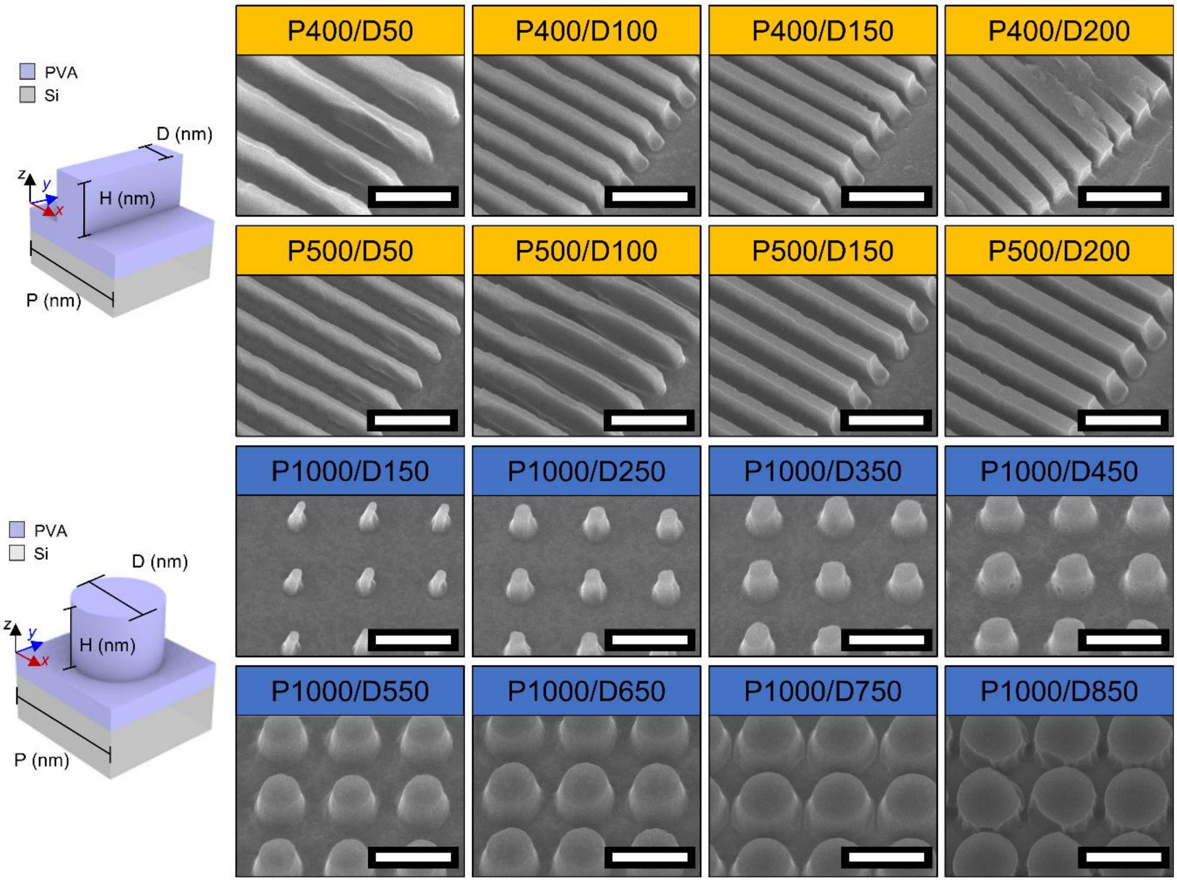

Supplementary Figure 6. Experimental resolution of polyvinyl alcohol (PVA) nanoimprint lithography (NIL). Tilted scanning electron microscope images of nanostructured arrays with various geometric parameters printed using the PVA resin. Scale bars: 1 μm.

Supplementary Note 9: Experimental confirmation of the non-toxic nature of the PVA metasurfaces

Since there are a few potentially harmful substances used in the fabrication of the soft mold that is used to replicate the polyvinyl alcohol (PVA) metasurfaces, we confirm that there is no trace of them left in the final one-step imprinted metasurfaces. First, it is worth mentioning that one of the coupling agents used to create the soft mold, toluene, is extremely volatile. This means that it almost instantly evaporates into the surrounding air, and therefore cannot be found in the final PVA metasurface. Another important substance that could be harmful to humans is silane coupling agent (Trichloro-1H, 1H, 2H, 2H-Perfluorooctyl-silane). The chemical structures of PVA and silane coupling agent are shown in Supplementary Fig. 7a. The elements fluoride (F) and chlorine (Cl) are only found in the harmful agent, therefore, to prove the absence of such toxic substances, we conduct energy-dispersive X-ray spectroscopy (EDS) analysis on both the soft mold and the one-step imprinted PVA Metasurfaces (Supplementary Fig. 7b). The results clearly show that the harmful elements are not transferred to the final PVA metasurfaces as there is no trace of them, proving their non-toxicity and potential for use in the food and biomedical industries.

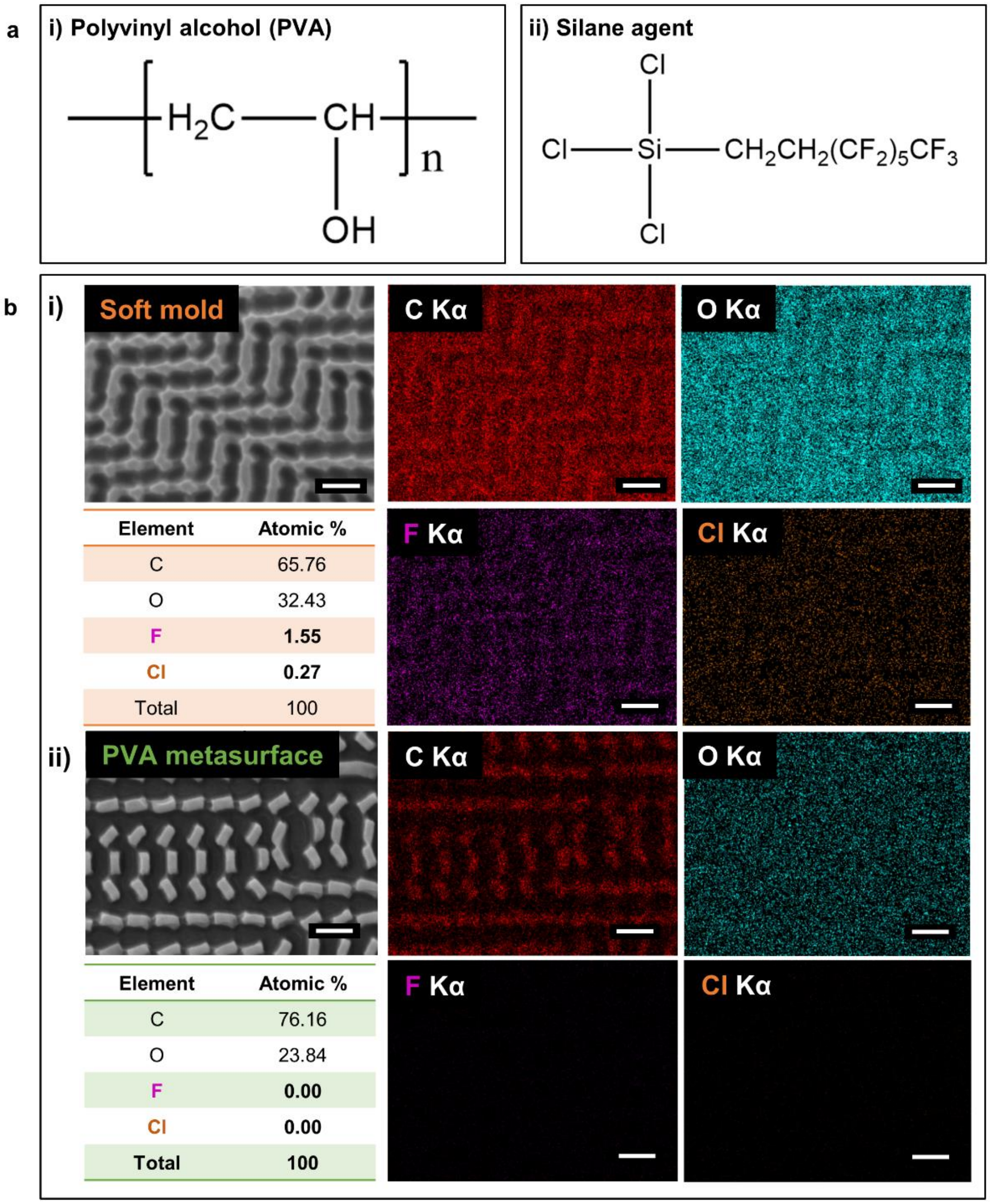

Element	Atomic %
C	65.76
O	32.43
F	**1.55**
Cl	**0.27**
Total	100

Element	Atomic %
C	76.16
O	23.84
F	**0.00**
Cl	**0.00**
**Total**	**100**

Supplementary Figure 7. **Experimental verification of the non-toxicity of the polyvinyl alcohol (PVA) metasurfaces. (a)** The chemical structures of (i) polyvinyl alcohol (PVA) and (ii) the silane agent (Trichloro-1H, 1H, 2H, 2H-Perfluorooctyl-silane). **(b)** Energy-dispersive X-ray spectroscopy (EDS) analysis of the (i) soft mold, and (ii) one-step imprinted PVA metasurface. Scale bars: 500 nm.

Supplementary Note 10: Reflectance spectra depending on geometry and sizes of PVA nanostructures

We verify different reflectance spectra can be obtained by changing filling ratio and geometry of subwavelength nanostructured polyvinyl alcohol (PVA). When the filling ratio is close to zero, its reflectance spectra is close to bare silicon dioxide (SiO_2) substrate (~4%). However, when the width of the nanocuboid or diameter of nanocylinder are larger than 200 nm, evident peaks appear due to impedance matching that satisfy antireflection conditions at certain wavelengths.

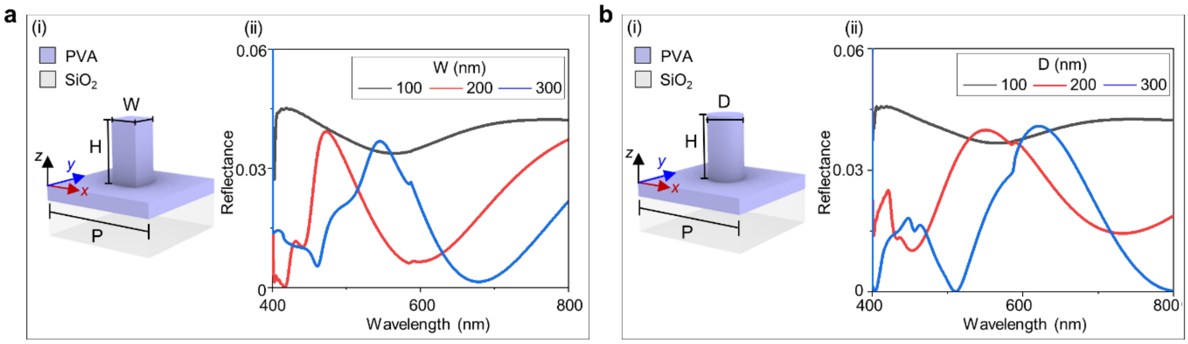

Supplementary Figure 8. Calculated reflectance spectra of structured polyvinyl alcohol (PVA). Different reflectance spectra are obtained when PVA is structured as a **(a)** nanocuboid and **(b)** nanocylinder. The periodicity *P* and the height *H* of the structures are 400 nm, and 400 nm, respectively. The width *W* and diameter *D* are varied from 100 nm to 300 nm in steps of 100 nm.

Supplementary Note 11: Theory of PB-phase

When left-circularly polarized light is incident on a rectangular shaped nanostructure, the output polarization of the light can be described using the Jones vector given by[4]

$$\frac{t_l + t_s}{2}\begin{bmatrix}1\\i\end{bmatrix} + \frac{t_l - t_s}{2}e^{i2\alpha}\begin{bmatrix}1\\i\end{bmatrix}, \tag{3}$$

where t_l and t_s denote the complex transmittance coefficient under linearly polarized light along the long and short axis, respectively; and α is the rotation angle of the rectangular shaped structure in the *xy*-plane. By manipulating α, the spatial phase of the converted circularly polarized light can be controlled to cover the entire 0-2π phase space.

Supplementary Note 12: PVA swelling simulations

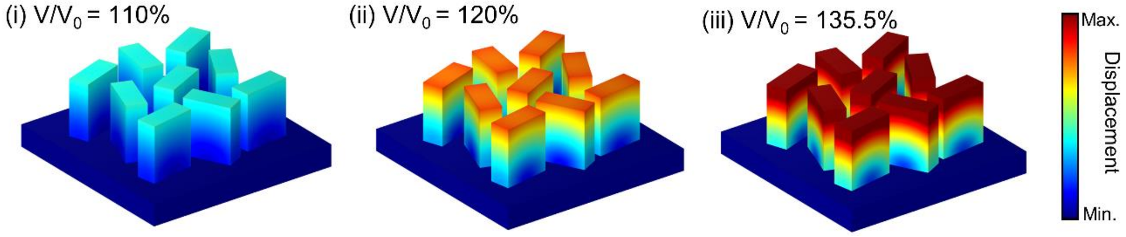

Supplementary Figure 9. Numerical simulations of the swelling of Meta A. Swelling simulation of Meta A when the origin volume is swollen by (i) 110%, (ii) 120%, and (iii) 135.5%.

To simulate the swelling of polyvinyl alcohol (PVA) nanostructures with regards to the relative humidity (RH), we model nine PVA structures on a substrate. The size of the structures is set to 350 × 200 × 500 nm3, to represent Meta A, and the rotation angles of blocks are set to arbitrary values between −90° ~ 90°.

The 3D swelling configuration of modeled structures is numerically calculated with the commercially available software, COMSOL Multiphysics version 5.6, using the solid mechanics and heat transfer modules. We conduct hygroscopic swelling simulations on the structures[5,6]. The substrate is considered to be rigid and the centers of the bottom of the PVA structures are fixed to the substrate. We assume that the material has an isotropic coefficient of hygroscopic expansion β [m3/kg], and β is increased from 0 until the volume swelling ratio (V/V_0) reaches 135%.” The maximum displacement is observed at the top corners of the meta-atoms, which means that any aggregation of the meta-atoms during swelling starts from the top of the meta-atoms, effectively destroying the structures.

Supplementary Note 13: Apparent unevenness in the reflected color

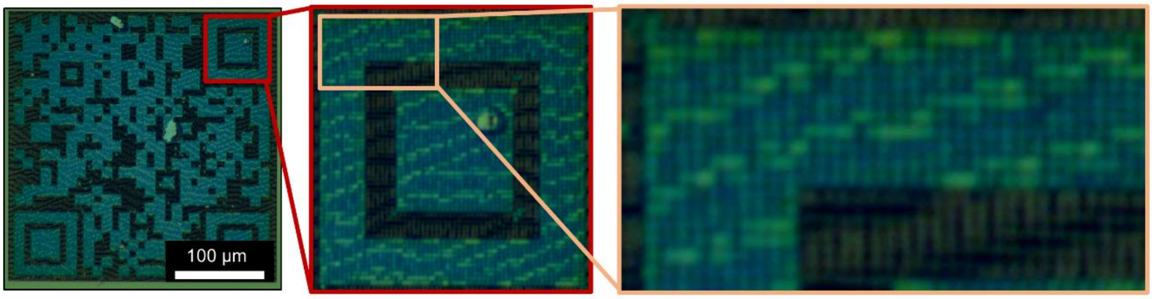

Supplementary Figure 10. Apparent color unevenness of the fabricated QR-code polyvinyl alcohol (PVA) metasurfaces.

In the images of the color prints taken using optical microscopy, a slight variation in color can be seen, especially under high magnifications. This is due to focusing on the near-field of the metasurface, which allows us to see the phase modulation due to the meta-atoms. To confirm this, the implemented phase map for the metasurface is shown in Supplementary Fig. 11. It is clear to see that the encoded phase map has the same wave-like patterns as is appear in the optical microscope images of the structural color prints.

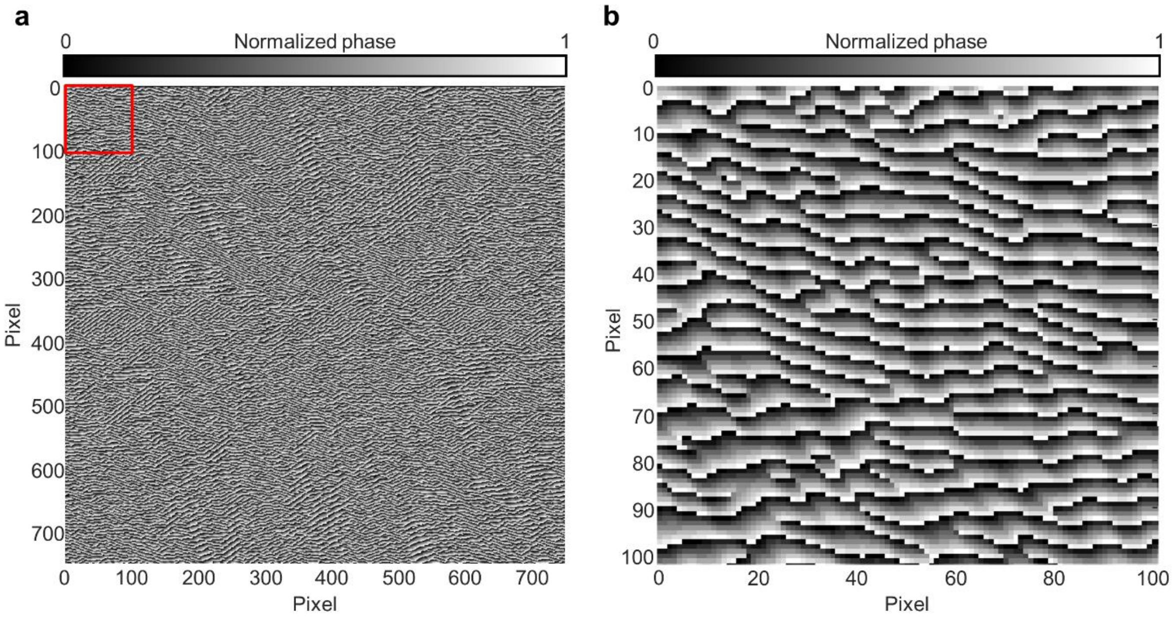

Supplementary Figure 11. Retrieved phase map for hologram generation. (a) Total phase map for QR-code metasurfaces, and **(b)** Magnified phase map images that is the red box of (a) the total phase map.

Supplementary Note 14: Measured relative humidity of human breath

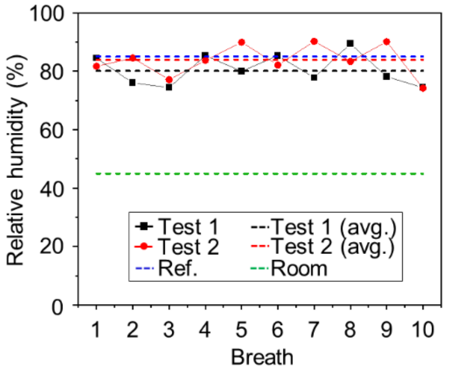

Supplementary Figure 12. Measured relative humidity (RH) of human breath. The RH of the room was measured to be 45%. Two different subjects breathed on the humidity sensor at a distance of 2.5 cm away for around 1.5 sec. The dots represent the maximum values of RH measured for each breath.

To confirm the value of RH that is achieved under the exhalation of a human breath, we measured the maximum RH using a commercial humidity meter (Testo, Testo 625). Two different researchers breathed on the meter at a distance of 2.5 cm away for around 1.5 sec, 10 times each, and the maximum RH reached was recorded (Supplementary Fig. 12). The RH of the room in which the experiment was conducted was measured to be 45%. The mean RH for subject 1 was measured to be 80.0%, while subject 2 was 83.9%. The measured values are comparable to the values quoted in the literature (85%)[7].

Supplementary Note 15: Calculated and measured reflectance spectra

The reflectance spectra of Meta A and Meta B are calculated under 45º rotated linearly polarized light incident illumination, and compared with the measured reflectance spectra of 150 × 150 μm^2 nanoimprinted polyvinyl alcohol (PVA) metasurfaces made up of either Meta A or Meta B under unpolarized white light illumination. H = 400 nm, P = 400 nm, $t_{a\text{-}Si:H}$ = 550 nm, and t_{Pt} = 10 nm are kept consistent throughout, while Meta A and Meta B both have L = 320 nm, and W = 200 and 90 nm, respectively. The calculated reflectance spectra of Meta A and Meta B show similar trends with measured spectra. We attribute the discrepancy of the reflectance intensity to geometric differences between the designed and fabricated meta-atoms, such as the rounded side walls.

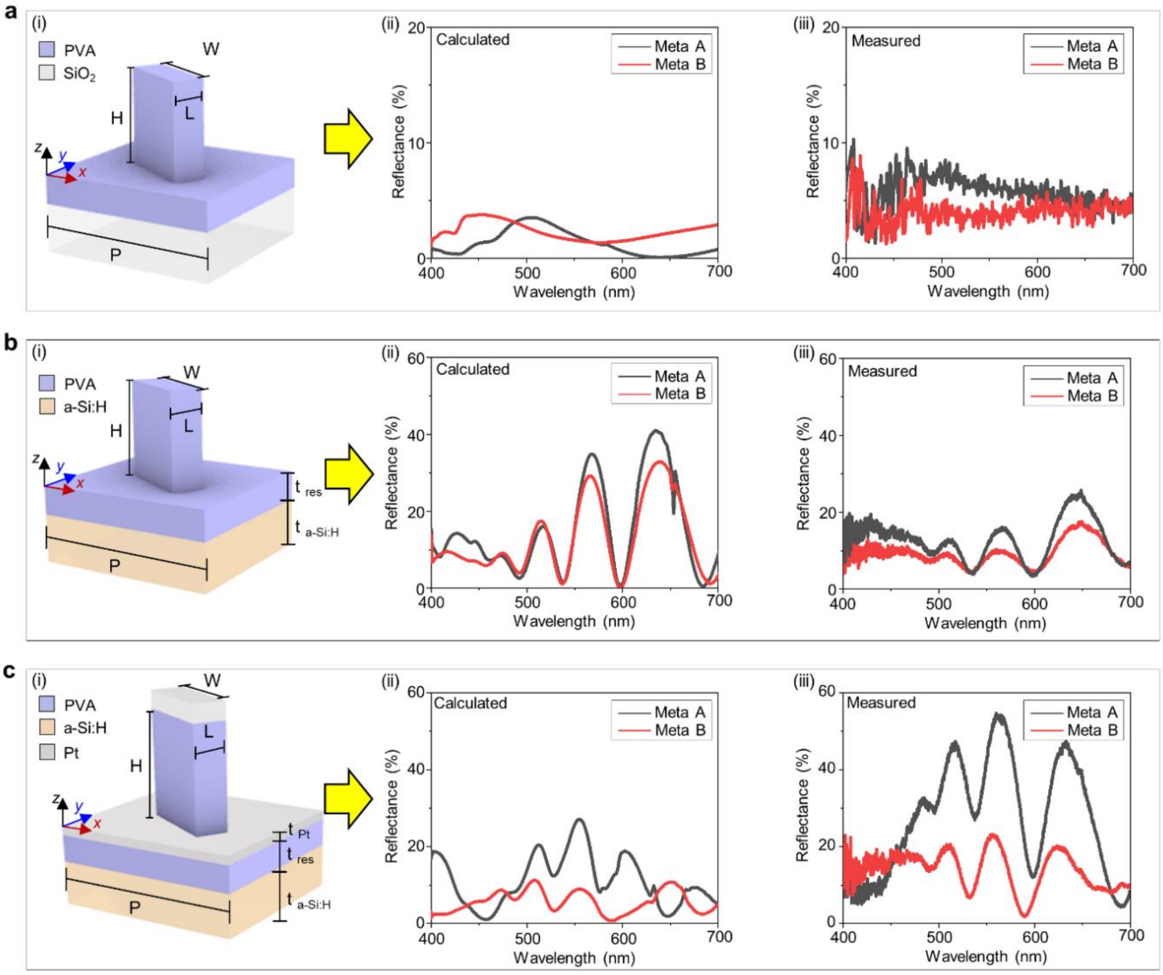

Supplementary Figure 13. **Calculated and measured reflectance spectra of Meta A and Meta B for each metasurface. (a)** Cross-sectional schematic of the meta-atom configuration **(b)** Calculated and **(c)** measured reflectance.

Supplementary Note 16: Measured absorption characteristics

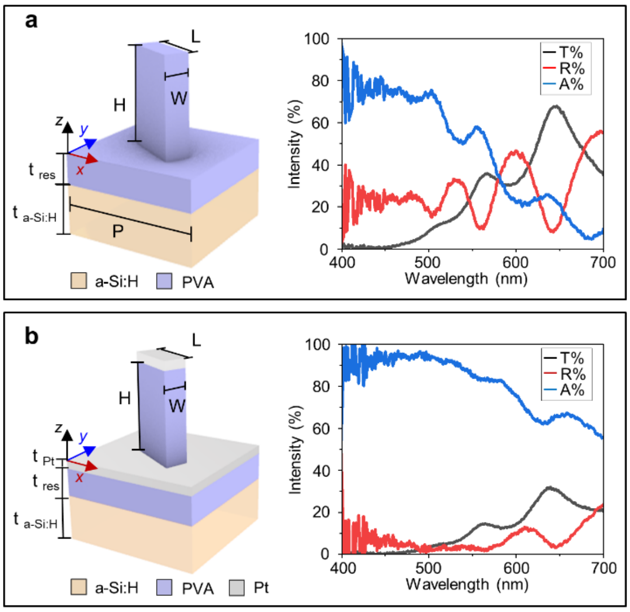

Supplementary Figure 14. The measured transmittance, reflectance, and absorptance spectra of Meta A. Measured spectra for **(a)** polyvinyl alcohol (PVA) metasurfaces made up of Meta A, and **(b)** the same metasurfaces with a 10 nm platinum (Pt) layer. Transmittance: black, reflectance: red, and absorptance: blue.

To determine the effect of adding the Pt coating to our PVA metasurfaces, we prepared metasurfaces made up Meta A and measured transmittance (T) and reflectance (R), and calculated the absorption (A) with and without the Pt coating. We measure T and R of the uncoated PVA metasurface using spectrometry (Supplementary Fig. 14a). After that, an ultra-thin, ~10 nm, Pt film is coated using an ion sputter at 20 mA for 60 s (Supplementary Fig. 14b), and the T and R are then measured again, A is calculated using A=1-R-T. The Pt coating causes around a 20-30% of extra absorption due to the high extinction coefficient of Pt in the visible regime, however, no plasmonic resonances are excited.

Supplementary Note 17: Increased noise in the holographic image due to high RH

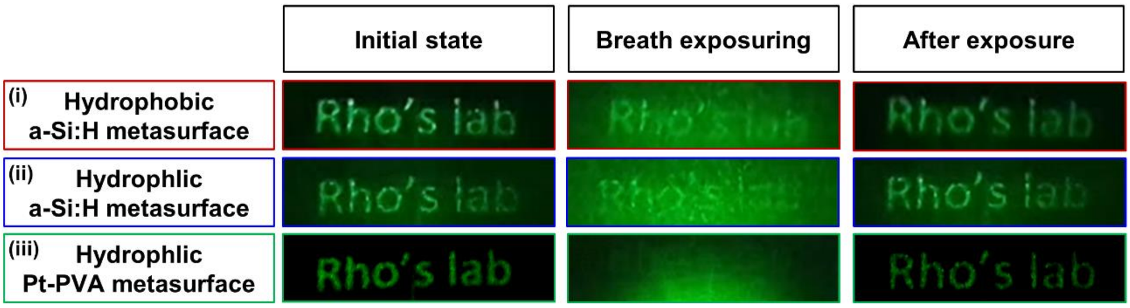

Supplementary Figure 15. The influence of high relative humidity (RH) conditions due to breathing on platinum (Pt)-coated polyvinyl alcohol (PVA) metasurfaces. Comparison of holographic images produced from the master mold made of (i) hydrogenated amorphous silicon (a-Si:H) with a hydrophobic coating, (ii) a-Si:H with a hydrophilic coating, and (iii) the hydrophilic Pt-coated PVA

When the Pt-coated PVA metasurfaces are exposed to high RH conditions using a breath, the holographic image is temporarily hidden, while the background noise is dramatically increased. To analyze the reason for the disappearance of the holographic image, we investigate the holographic images produced by the a-Si:H master mold. First, we treat it with a hydrophobic coating to help repel any water vapor, and then we treat with a hydrophilic coating, to emulate the hydrophilicity of the PVA metasurfaces. All three samples are exposed to the same high RH conditions using a breath and the holographic images are captured (Supplementary Fig. 15). It can be seen that during exposure to a breath, all three samples demonstrate a dramatically increased background noise to the holographic image. This can be attributed to water molecules forming on the metasurfaces which promote local scattering of the light. Although the background noise is increased, both holographic images from the a-Si samples are visible, whereas the Pt-coated PVA metasurface temporarily displays no discernable image. We attribute this to the reduction in the refractive index of the PVA meta-atoms as well as increased scattering due to absorbing water molecules.

Supplementary Note 18: Measurement of the decryption threshold for the Pt coated PVA metasurfaces

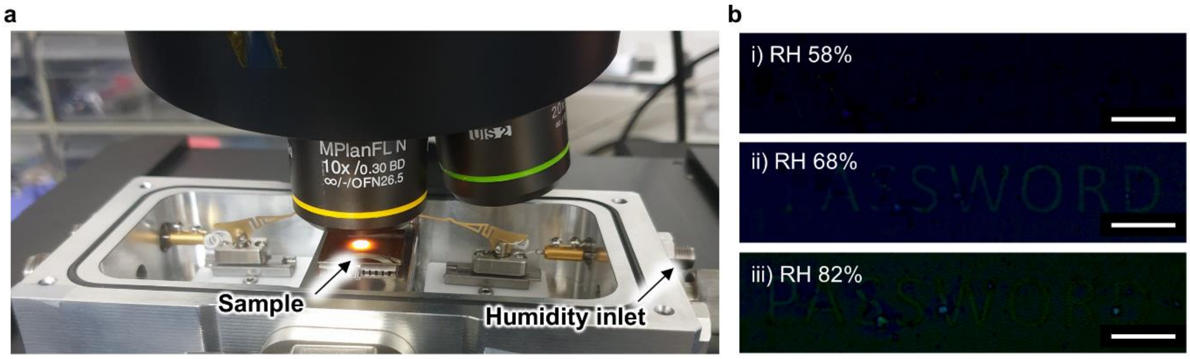

Supplementary Figure 16. Measurement of the decryption threshold. (a) Measurement setup to quantify threshold of decryption. The chamber is uncovered in the figure for clarity, but it is covered in the experiment. **(b)** Optical images are obtained at i) Relative humidity (RH) 58%, ii) RH 68%, and iii) RH 82%. Scale bar: 50 µm.

The threshold RH to decrypt the hidden structural color image in the platinum (Pt)-coated metasurfaces is measured using a humidity sensor implanted optical microscopy setup (Supplementary Fig. 16a). The sample had already been exposed to human breath 100 times to validate the repeatability of the optical encryption system. The RH is increased in the chamber by inserting N_2 gas to gradually increase the RH from 58% to 82%. The image is decrypted at RH 68%, and continues to be visible until around RH 82% (Supplementary Fig. 16b), which correlates to the RH of human breath.

Supplementary Note 19: Robustness of the PVA metasurfaces

We conducted experiments to confirm the robustness of the polyvinyl alcohol (PVA) metasurfaces in response to exposure to high temperatures by baking them at 70 °C for 72 h. From the scanning electron microscope (SEM) images (Supplementary Fig. 17), it is clear to see that the meta-atoms geometries are maintained without any distinguishable defects. This is further confirmed through the holographic image, which is reproduced successfully after the exposure to high temperatures. Furthermore, we confirm the performance of thermal-annealed sample by exposing it to 1.5 sec of exhaled breath 100 times. The SEM images after 20 and 100 exposures show no obvious defects and the holographic images are reproduced successfully, proving the potential for the use of our PVA metasurfaces in everyday room conditions which have temperatures and relative humidity (RH) that are generally lower than 70 °C and 80%, respectively.

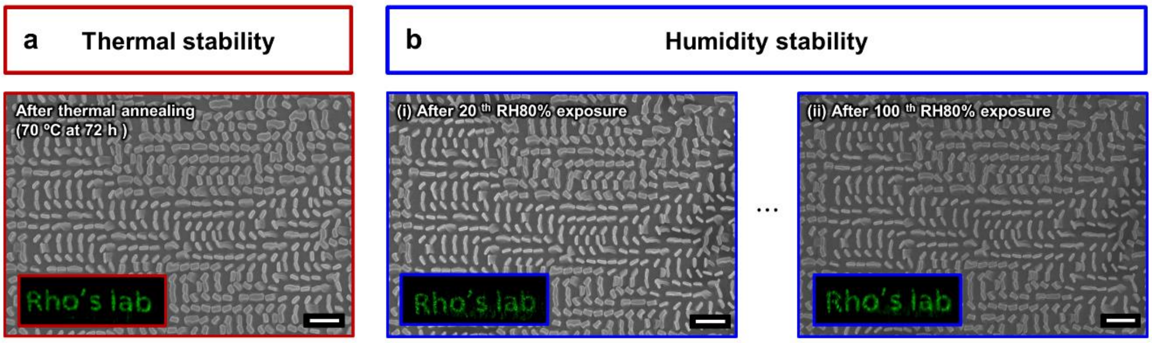

Supplementary Figure 17. Robustness of the polyvinyl alcohol (PVA) metasurfaces. (a) scanning electron microscope (SEM) image of thermally annealed reversible PVA metasurfaces after exposure to 70 ˚C for 72 h. Inset: Holographic image. **(b)** SEM images of the annealed sample after (i) 20, and (ii) 100 exposures to 80% relative humidity (RH). Insets: Holographic images. Scale bars: 1 μm

References

1. Larena, A., Millán, F., Pérez, G. & Pinto, G. Effect of surface roughness on the optical properties of multilayer polymer films. *Appl. Surf. Sci.* **187**, 339–346 (2002).
2. Markel, V. A. Introduction to the Maxwell Garnett approximation: tutorial. *J. Opt. Soc. Am. A* **33**, 1244–1256 (2016).
3. Schaper, C. D. & Miahnahri, A. Polyvinyl alcohol templates for low cost, high resolution, complex printing. *J. Vac. Sci. Technol. B Microelectron. Nanom. Struct.* **22**, 3323 (2004).
4. Kim, J. *et al.* Geometric and physical configurations of meta-atoms for advanced metasurface holography. *InfoMat* **3**, 739–754 (2021).
5. Ehrenhofer, A., Elstner, M. & Wallmersperger, T. Normalization of hydrogel swelling behavior for sensoric and actuatoric applications. *Sen. Actuators B Chem.* **255**, 1343–1353 (2018).
6. Yang, Y. & Zhao, H. Water-induced polymer swelling and its application in soft electronics. *Appl. Surf. Sci.* **577**, 151895 (2022).
7. Dai, J. *et al.* In Situ Preparation of Porous Humidity Sensitive Composite via a One-Stone-Two-Birds Strategy. *Sens. Actuators B Chem.* **316**, 128159 (2020).
