# Supplementary material for: Tunable metasurfaces via the humidity responsive swelling of single-step imprinted polyvinyl alcohol nanostructures
Source: Nat Commun. 2022 Oct 21;13:6256. doi: 10.1038/s41467-022-32987-6 (PMC9587293; doi:10.1038/s41467-022-32987-6)
Supplement: Supplementary file 3 — Description to Additional Supplementary Information [file 41467_2022_32987_MOESM3_ESM.pdf]

Legend for Supplementary Movie:

Supplementary Movie 1. PVA nanoprint destruction under high humidity

Supplementary Movie 2. Nanoprint decryption and destruction due to high humidity

Supplementary Movie 3. Pt-coated PVA metasurface for reversible metaholography and nanoprint encryption

(hologram demonstration)

Supplementary Movie 4. Pt-coated PVA metasurface for reversible metaholography and nanoprint encryption

(nanoprint demonstration)
